# A Rare Case of Pancreatoblastoma in a Pediatric Patient

**DOI:** 10.7759/cureus.6779

**Published:** 2020-01-27

**Authors:** Moaffaq Mahdi, Mazen Abu Alnasr, Bassel A Almehman, Safi Nassan, Soliman Bin Yahib

**Affiliations:** 1 Medicine, King Saud Bin Abdulaziz University for Health Sciences, Jeddah, SAU; 2 Medicine, King Saud Bin Abdulaziz for Health Sciences, Jeddah, SAU; 3 General Surgery, King Saud Bin Abdulaziz University for Health Sciences, Jeddah, SAU; 4 Pediatric Surgery, King Abdulaziz Medical City, Jeddah, SAU

**Keywords:** • pancreatic tumors, pancreatoblastoma, blastoma, pancreas

## Abstract

Pancreatoblastoma (PB) is a rare pancreatic neoplasm which arises when a group of pancreatic cells start to go through uncontrollable growth. The diagnosis of PB is challenging due to its vague symptoms. The initial diagnosis is made by imaging, afterwards the management is usually by resection of the tumor with or without chemotherapy which depends on the size and grade of the tumor. We report a case of a nine-year-old girl who was diagnosed with pancreotoblastoma and underwent complete resection with chemotherapy.

## Introduction

Pancreatic tumors are conditions which arise when a group of pancreatic cells start to go through uncontrollable growth [1]. Pancreatoblastoma (PB) is a rare malignant neoplasm that originates from primitive cells [2]. With an incidence of around 0.004 per 100,000 cases every year, PB is one of the most common pancreatic tumors [3]. In the pediatric age group, the diagnosis of PB is mostly incidental. The challenging part in diagnosing PB comes from the nonspecific symptoms that depend on the location and the features of the neoplasm [4]. PB produces alpha-fetoprotein (AFP) only in 30% of the patients [2]. On the other hand, PB has similar genetic type to other pediatric blastomas, for example, nephroblastoma, and hepatoblastoma all show the same allelic loss on the maternal copy of chromosome 11p [5].

Pancreatoblastoma management varies according to the stage. Until now around 200 cases were reported, and the surgical intervention plays a significant role in attaining complete remission [6]. Prognosis depends on the tumor grade, for example, low grade tumors tend to be resected which has good prognosis. On the other hand, having partially resectable or metastatic tumors shows a poor prognosis [7].

## Case presentation

A nine-year-old girl complained of mild abdominal pain in the last three years. The patient had a history of several admissions with diagnosis of urinary tract infection. Before one month, the abdominal pain started to increase in intensity and the mother started to notice loss of weight. She was diagnosed with hepatoblastoma and was referred to our hospital for further management.

**Figure 1:**
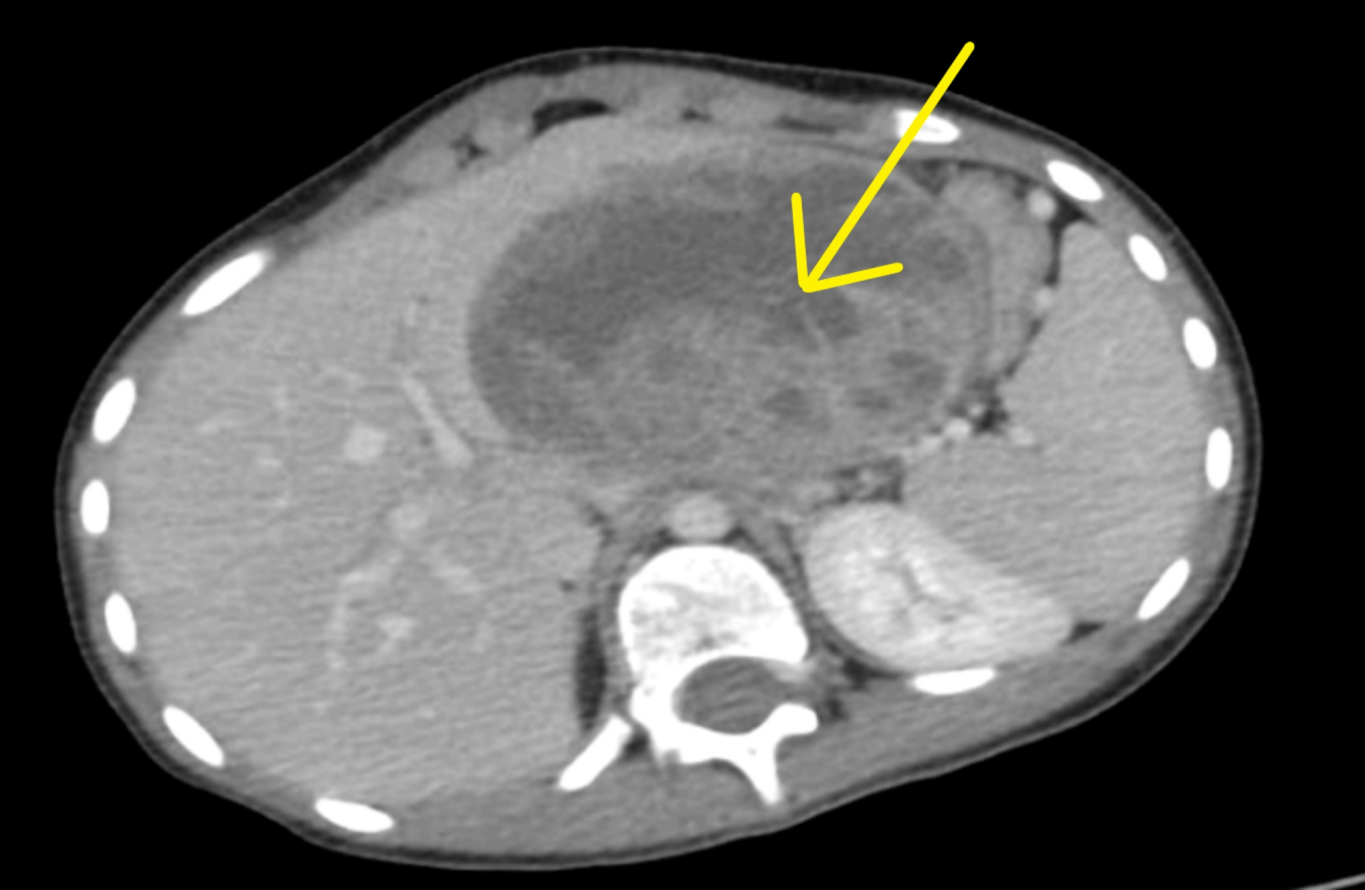
CT scan showing a huge lesion measuring 6.2 x 8.7 x 9.4 in diameter.

On admission the clinical examination showed underweight looking girl; chest and cardiovascular examination was unremarkable. Abdominal examination showed a mass occupying most of the left upper quadrant region. In addition, laboratory tests showed abnormal tumor markers of alpha-feto-protein (AFP) 45.6 IU/mL (normal 0.0-35.0) and cancer antigen 125 (CA 125) of 44.1 IU/mL (normal 0.0-35.0). Chest CT scan was done and showed a huge heterogeneously enhancing epigastric lesion measuring 6.2 x 8.7 x 9.4. Multiple mesenteric enlarged lymph nodes, the largest of which measured 1 cm x 1.7 cm. The lesion was probably arising from the liver; however, it is inseparable from the pancreas (Figure 1).

The patient underwent laparotomy to obtain biopsy for this lesion. During surgery the tumor was adherent to the left lobe of the liver segments II and III; the adherence was under the surface which made it very difficult to distinguish if the tumor is arising from the liver or invading it. The histopathology report showed fragments of dense fibrous tissue with hyper-cellular proliferation of small round blue cells with mild to moderate nuclear pleomorphism.

**Figure 2:**
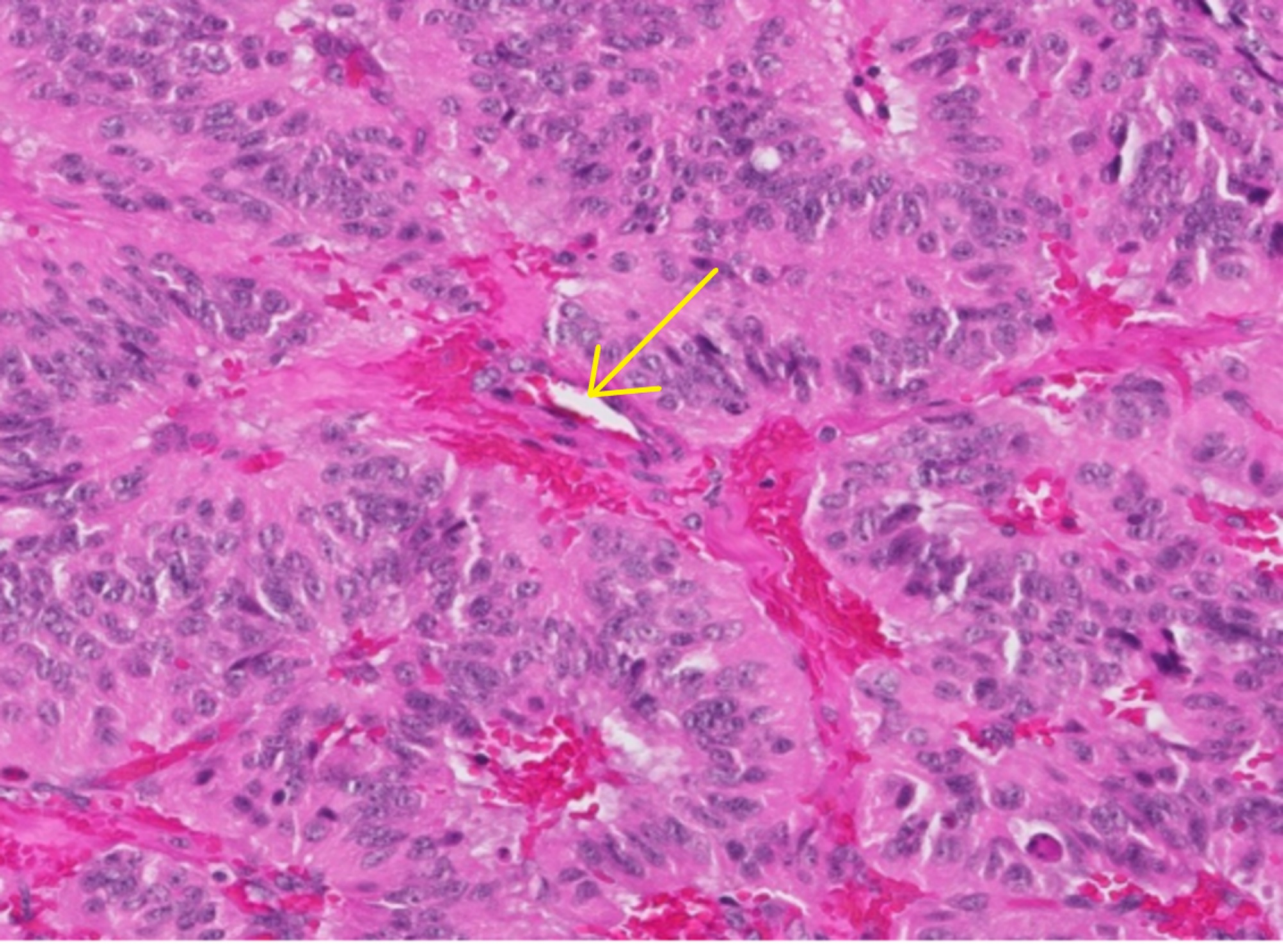
Fragments of dense fibrous tissue with hyper-cellular proliferation of small round blue cells with mild to moderate nuclear pleomorphism.

The cells were arranged in sheets forming numerous cords and rossettes with focal areas of acinar type architectural differentiation. No papillary cords were identified. None of the rossettes have central vessels. There were areas of syncytial arrangement of cells with more abundant pink cytoplasm which were consistent with squamoid nests. The nests were vaguely circumscribed, however, did not demonstrate any keratinization. Foci of foamy cells were identified, but perivascular hyalinization was not identified. No cholesterol clefts were identified or eosinophilic globules (Figure 2).

**Figure 3:**
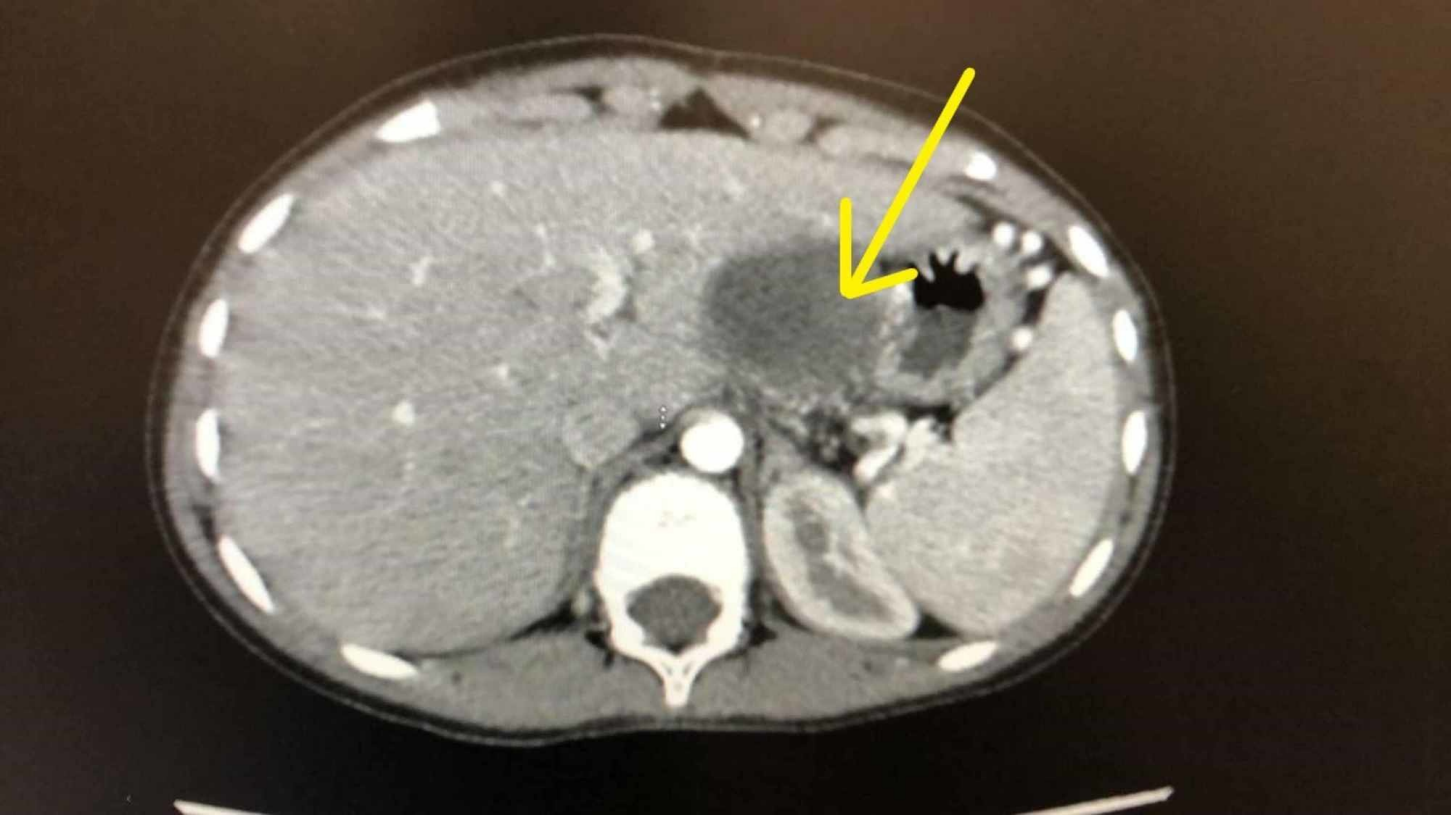
CT scan showing the lesion after chemotherapy, measuring 3.4 x 3.3 x 3.5 in diameter.

Immunohistochemical stains demonstrated diffused cytokeratin A1, A3 positivity, prominent multifocal synaptophysin positivity and focal chromogranin positivity. Multiple foci are positive for CD10. ER and PR are negative. AFP negative, S100 negative. Scattered mitosis approximately 2-3 10/HPF are present. The biopsy confirmed the diagnosis of PB stage III. The patient was started on chemotherapy, and she received doxorubicin. And she was on PLADO protocol. After six cycles of chemotherapy a CT scan showed significant changes with decrease in size measuring 3.4 x 3.3 x 3.5. after that the patient underwent subtotal pancreatectomy and resection of tumor (Figure 3). The patient tolerated the surgery well and was discharged home after eight days.

## Discussion

Although PB is one of the common pancreatic tumors, still pancreatic tumors in children are considered rare. Since Becker [8] reported the first case of PB in infancy, there are only about 200 cases reported worldwide. Furthermore, PB tumors arising from the tail of the pancreas are even more rare. This rarity gives lack of an international standardized treatment strategy for PB. Multiple cases of PB have been treated with preoperative chemotherapy. However, these cases present usually with large masses and/or metastases. Adjuvant chemotherapy postoperatively has not been frequently used. Instead, some cases used radiotherapy postoperatively. A small PB without metastases can be surgically removed without pre- or postoperative chemotherapy in order to achieve complete remission.

Pancreatoblastoma is usually incidentally found because most of the time it is asymptomatic. In a study by Xu et al. [9] where they reported three cases of PB, two of the three cases were only identified after finding an abdominal mass in rather a casual abdominal examination.

As per our case, the patient presented with multiple attacks of abdominal pain which gave rise to multiple differential diagnoses, one of which was PB. An abdominal mass was only identified radiologically. Moreover, most reported cases presented with abdominal pain and PB was only diagnosed incidentally via imaging. According to the size and nonmetastases of our case, chemotherapy of PLADO protocol was used. For instance, one of the reported cases used neo-adjuvant chemotherapy to regress the metastatic lesions and the tumor [10]. Similarly, in our case, after PLADO protocol, the tumor was markedly shrunk which made it easier to get resected. In conclusion, we faced a case of simple PB which was fully resected and the patient achieved complete remission.

We recommend commencing a multicenter clinical trial in order to have sufficient data to construct a standardized treatment protocol for PB cases.

## Conclusions

Pancreatoblastoma is a rare malignant neoplasm that originates from primitive cells. With an incidence of around 0.004 per 100,000 cases every year. PB diagnosis is challenging due to its nonspecific clinical manifestations. Management of this tumor is mainly by surgical resection with or without chemotherapy which depends on its size and stage.
